# Supplementary figures and images for: Centromere Protein F Is a Potential Prognostic Biomarker and Target for Cutaneous Melanoma
Source: Biomedicines. 2025 Mar 25;13(4):792. doi: 10.3390/biomedicines13040792 (PMC12024980; doi:10.3390/biomedicines13040792)

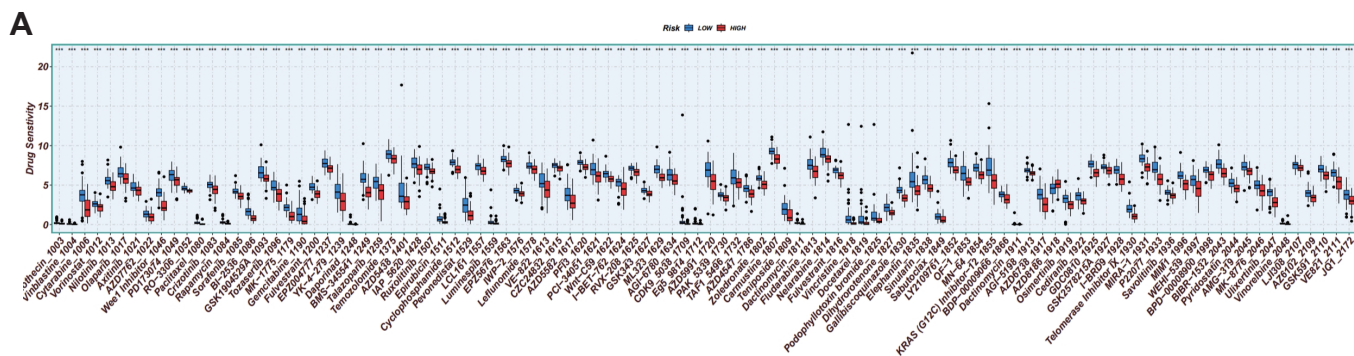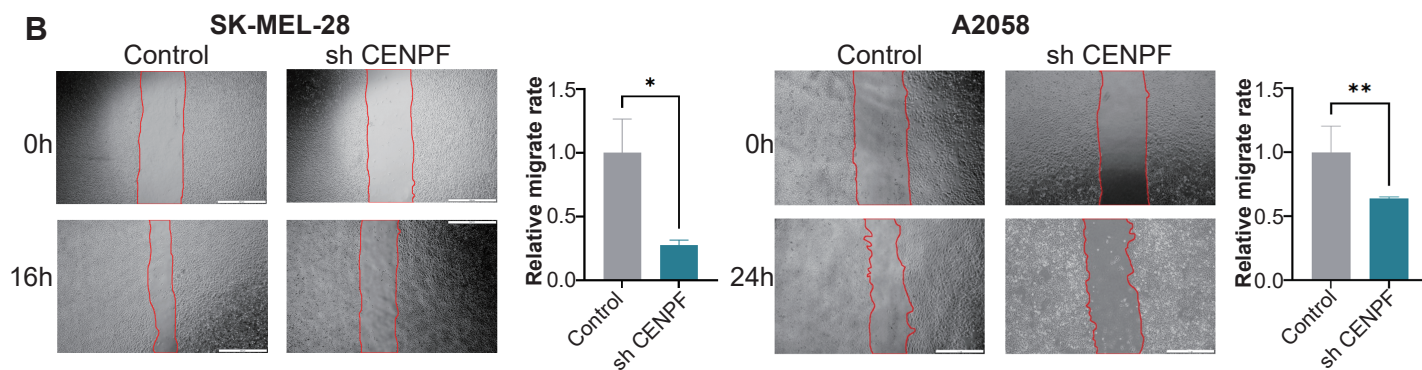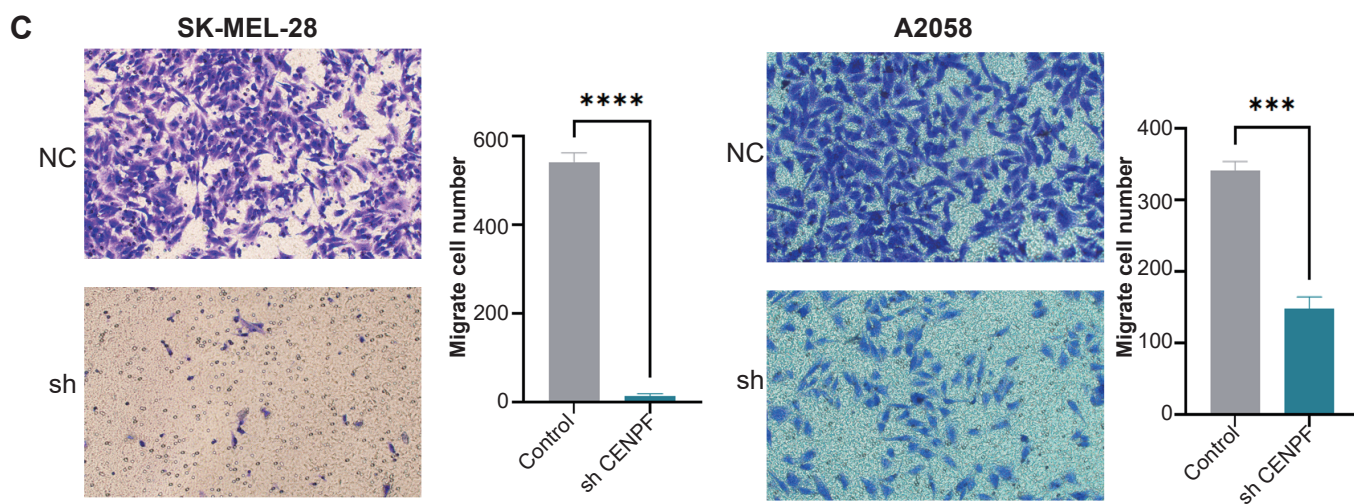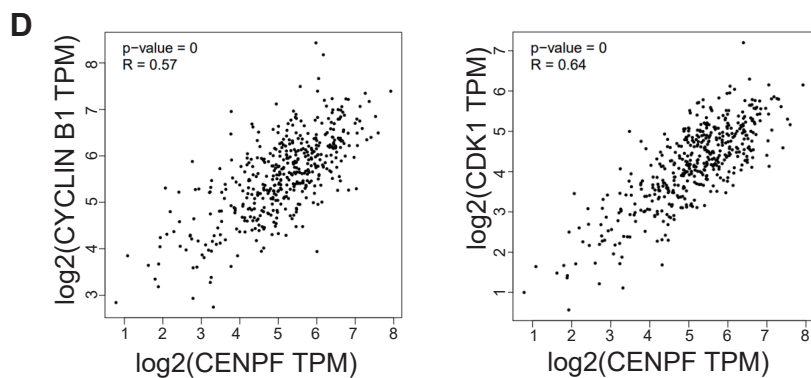

Supplement: Supplementary file 1 [file biomedicines-13-00792-s001.zip › Figure S1.pdf]

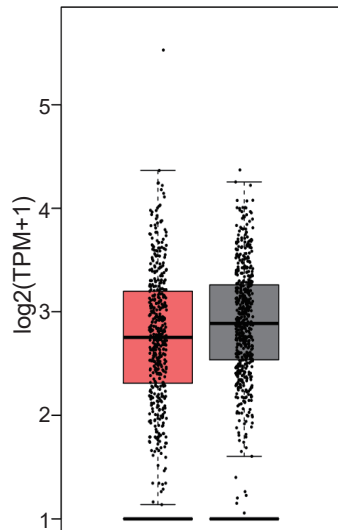

**E2F2**

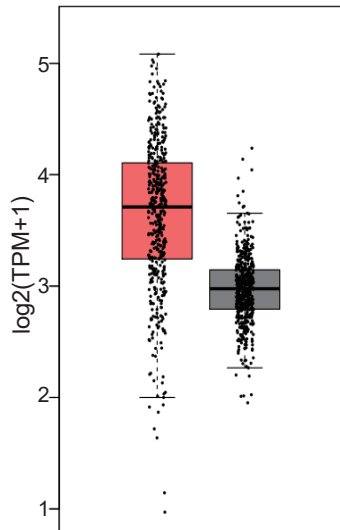

**E2F7**

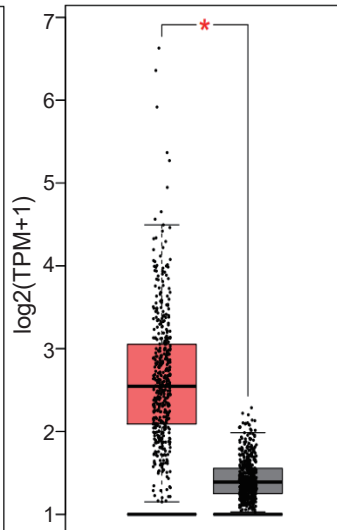

**E2F6**

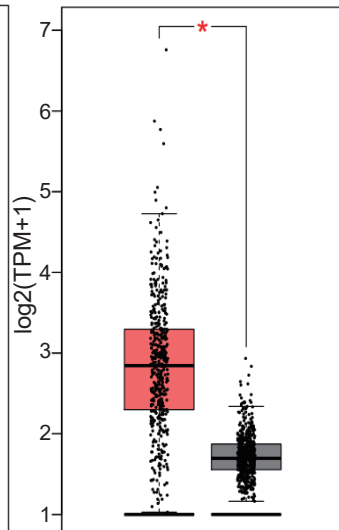

**MYBL1**

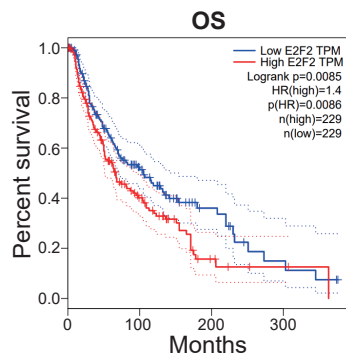

**OS**

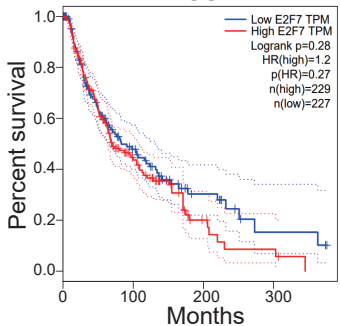

**OS**

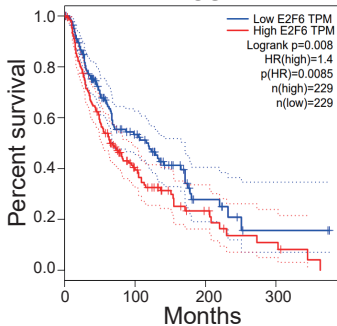

**OS**

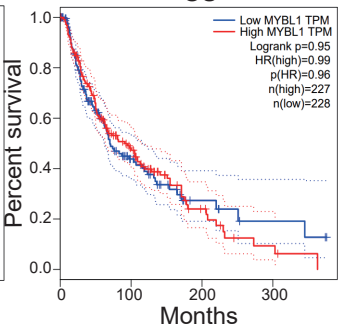

**OS**

Supplement: Supplementary file 1 [file biomedicines-13-00792-s001.zip › Figure S2.pdf]
